# Neuromuscular control of ankle and hip during performance of the star excursion balance test in subjects with and without chronic ankle instability

**DOI:** 10.1371/journal.pone.0201479

**Published:** 2018-08-13

**Authors:** Hatem Jaber, Everett Lohman, Noha Daher, Gurinder Bains, Abhay Nagaraj, Prajakta Mayekar, Manali Shanbhag, Mansoor Alameri

**Affiliations:** 1 Department of Physical Therapy, School of Allied Health Professions, Loma Linda University, Loma Linda, California, United States of America; 2 Department of Allied Health Studies, School of Allied Health Professions, Loma Linda University, Loma Linda, California, United States of America; University of Illinois at Urbana-Champaign, UNITED STATES

## Abstract

**Background/Purpose:**

Ankle sprains are common and potentially disabling musculoskeletal injuries that often lead to chronic ankle instability (CAI). CAI has been linked to impairments in postural and neuromuscular control; however, inconsistent findings have been reported. Individuals who experience a lateral ankle sprain, but do not develop instability, termed copers, may adapt different neuromuscular control strategies after injury. This study aimed to compare postural control and electromyographic (EMG) activity of hip and ankle muscles during the performance of the Star Excursion Balance Test (SEBT) in subjects with and without CAI.

**Method:**

48 participants were classified into three groups (16 control, 16 copers, 16 CAI) based on ankle sprain history and Cumberland Ankle Instability Tool score. Outcome measures included normalized reach distance, center of pressure (COP), and integrated EMG activation of gluteus medius (Gmed), gluteus maximus (Gmax), tibialis anterior (TA), and peroneus longus (PL) during each reach direction of SEBT.

**Results:**

Compared to copers and controls, CAI group demonstrated significantly diminished postural control (reach distance and COP measures, p< 0.05) and less EMG activity of TA during the anterior direction (CAI: 33.1% ± 10.1% versus copers: 44.8% ± 12.7% versus controls: 51.7% ± 8.4%, p<0.01) and Gmax in the posterolateral direction (CAI: 25.6% ± 9.4% versus copers: 37.5% ± 13.8% versus controls: 40.2% ± 17.2%, p = 0.011).

**Conclusion:**

Alteration in proximal and distal muscle activity appears to negatively affect postural control and quality of movement, which may lead to prolonged functional impairments. Hence, implementing hip and ankle muscle exercises in the rehabilitation of ankle instability might benefit these patients.

## Introduction

The ankle joint is the second most commonly injured part of the body during sports, with lateral ankle sprains (LAS) being one of the most common musculoskeletal injuries among physically active individuals [1,2]. LAS account for approximately 25% to 30% of all sport-related injuries [2], with an incidence rate of 2.15 per person-year in the United States [3]. Although symptoms associated with LAS usually resolve quickly, it is estimated that approximately 40% of individuals who encounter an initial ankle sprain will develop persisting symptoms including pain, subjective instability or “giving way”, loss of function, and repetitive ankle injuries leading to a longstanding ankle dysfunction known as chronic ankle instability (CAI) [4].

It has been reported that postural control is altered after an acute lateral ankle sprain [5]. Evidence has suggested that CAI is often associated with poor postural control [5,6]. Impairments of postural control are usually thought to be the consequences of proprioception and neuromuscular control (NMC) deficits that occur after ligamentous injury [6]. Neuromuscular deficits, specifically alterations in the lower extremity muscle activation patterns have been considered as a major contributing factor to the impairments that affect stability and perceived function in patients with CAI [7]. Patients with CAI have demonstrated altered NMC strategies during functional activities [8,9]. During walking, patients with CAI exhibited an increase in peroneus longus (PL) activity prior to initial contact (IC) as opposed to healthy individuals who did so after IC [8]. Whereas, during a unipedal drop jump, patients with CAI demonstrated less anticipatory PL muscle activity compared to healthy controls [9]. These alterations in motor control were suggested as possible contributors to the inversion injuries in this population [9]. NMC also has been compared between individuals with CAI and those who have experienced ankle sprains but did not develop CAI [10,11]. This group of individuals are defined as copers [11]. When compared to CAI patients and healthy controls, copers had an increase in PL activity during jump landing [10] and tibialis anterior (TA) activity during the pre- and post-touchdown phases of gait [11]. The authors concluded that copers might have acquired these adaptive strategies as a protective mechanism to prevent reinjury [10,11]. However, limited evidence exists to support this conclusion.

While damage to the peripheral mechanoreceptors that provide proprioceptive input may result in altered NMC [12], disruptions in the central pathways for NMC are also thought to occur following the injury [13,14,15], suggesting that deficits associated with CAI may be the consequences of both peripheral and centrally mediated alterations in NMC. However, limited information exists about these alterations in this population. Most of the previous research that studied this phenomenon has focused primarily on NMC impairments at the injured ankle joint complex. Although this is a viable means for providing answers regarding changes that occur at single joint neural centers, recent research has identified disruption in proximal joints neuromuscular activation patterns in patients with CAI [16,17]. Webster and Gribble [16] reported decreased gluteus maximus (Gmax) activity in those with CAI during a single leg rotational squat exercise. Patients with CAI have also displayed a delay in onset of muscle activation and less anticipatory activation in muscles around the ankle, knee, and hip during a transition from bilateral to unilateral stance, which might indicate an involvement of multiple neural pathways [18]. However, despite previous findings, it is still unknown whether these alterations are responsible for the deficits in postural control in this population.

Postural control is an essential requirement for all motor tasks [19]. It can be classified as either static or dynamic [20]. Static postural control is the ability to maintain a stable base of support with minimal movement, whereas dynamic postural control is the capability of maintaining a stable base of support while completing a specific movement [20]. Different testing protocols have been used to quantify postural control in patients with CAI, including the Star Excursion Balance Test (SEBT) [21,22]. The SEBT has been deemed a reliable and valid clinical test in distinguishing dynamic postural control differences between subjects with and without stable ankles [23,24]. Postural control during the SEBT is reflected by the reach distance in 8 different directions, where a greater postural control is typically indicated by an increase in reach distance while maintaining a stable unilateral base of support [23]. Although numerous studies have used this test to assess postural control in patients with CAI, there has been less investigation regarding muscles activation necessary to complete the SEBT in patients with CAI. Gribble et al [22] reported that patients with CAI had increased postural control deficit as measured by the SEBT when compared to healthily controls. Though muscles activity was not measured in their study, the authors suggest that proximal muscles activation may have been altered, resulting in decreased knee and hip flexion angles and subsequent decreases in reach distance. Electromyographic (EMG) data, however, was not collected in the study, which limited their ability to fully identify the NMC strategies utilized by these patients.

Proximal motor control at the hip, specifically the gluteus medius (Gmed) and Gmax, is crucial for maintaining postural stability during weight bearing activities [25,26], and might be affected in CAI. Previous research has extensively focused on alterations in the ankle musculature with less emphasis on the activity of hip muscles necessary to complete a functional task in CAI patients. Hence, more research is needed to further understand the effects of proximal and distal neuromuscular alterations on postural stability in this population. Simultaneous analysis of the ankle and hip muscles EMG activation as well as dynamic postural control during the performance of the SEBT in subjects with and without CAI (copers & healthy) have not been previously examined. Examining the activity of the ankle and hip muscles during the performance of a dynamic task can provide more insight into the neuromuscular strategies utilized by these individuals to maintain postural stability. Additional knowledge regarding the interaction between hip and ankle muscle function during this activity may enhance the current understanding of CAI and help in customizing rehabilitation protocols that specifically target and improve patient outcomes. Therefore, the purpose of this study was to compare dynamic postural control and EMG activity of the TA, PL, Gmed, and Gmax muscles during the performance of the SEBT in subjects with and without CAI.

## Materials and methods

### Participants

A sample of forty-eight physically active volunteers (23 males, 25 females) participated in this study. All subjects read and signed an informed consent approved by the Institutional Review Board of Loma Linda University prior to participation. All subjects met the following inclusion criteria: 1) were between 18 and 35 years of age; 2) had a history of at least 1 significant lateral ankle sprain to the same side that resulted in pain and loss of function of more than one day (for CAI and coper groups); 3) sprain occurred not less than 12 months ago with no complaint of disability and/or giving way episodes since the injury (for copers); 4) had a history of at least 2 episodes of ‘giving way’ in the past 6 months (for CAI group); 5) had no history of ankle sprains (for the control group); and 6) participate in physical activity for at least 90 minutes each week [27]. Subjects were excluded if they reported: 1) bilateral ankle instability; 2) a history of neuromusculoskeletal or vestibular disorders; 3) previous lower limb surgeries; 4) trauma to the lower limbs for at least 3 months prior to the study; 5) physiotherapy within the last 3 months or current participation in supervised physical rehabilitation; and 6) consumed drugs or alcohol within 24 hours prior to testing that could interfere with performance.

Perceived ankle instability was assessed using self-reported questionnaires that included the Cumberland Ankle Instability Tool (CAIT) (minimum score 0, maximum score 30) and the Ankle Instability Instrument (AII). The CAIT is valid and reliable in assessing the perceived symptoms of ankle instability [28], and the combination of the two instruments (the AII and CAIT) was reported to be most accurate in classifying CAI [29]. Subjects were classified as having CAI if they scored 24 or less on CAIT, which was confirmed with the AII (answered ‘yes’ to at least five questions, including question 1) [29]. Scores of 28 or higher were defined as functionally stable ankles (copers or controls). Subjects who scored between 24 and 28 were excluded from the study to control for any potential effect on the results. Subjects were then placed in CAI, coper, or control group based on the history of lateral ankle sprain and the presence/absence of ankle instability. All measurements were taken on the injured limb for the CAI and copers groups, and on the dominant limb for the control group, which was defined as the limb used to kick a ball.

### Instrumentation

#### Postural control

**Fig 1:**
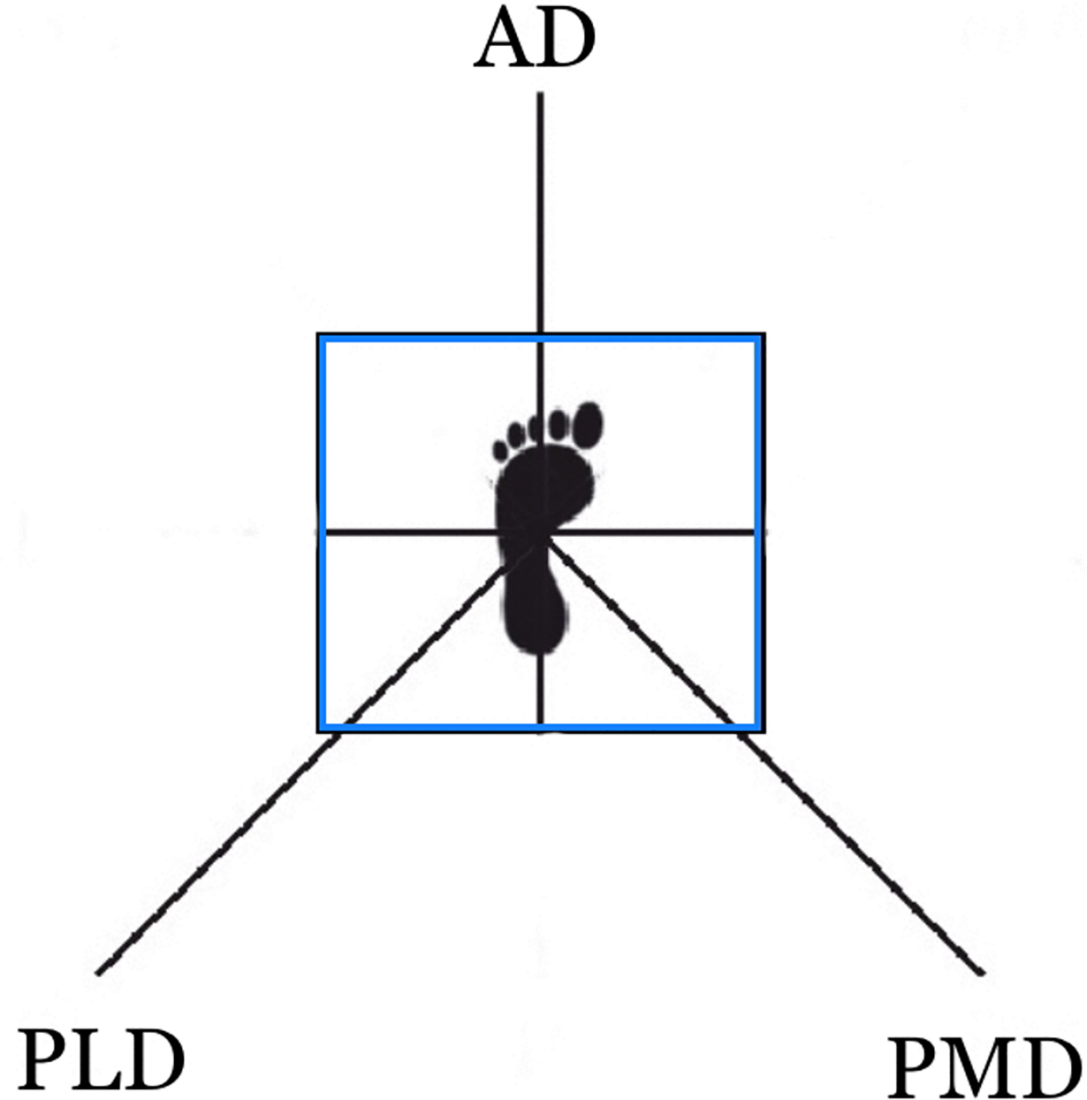
Star Excursion Balance Test for a left test leg. The box represents the force platform. The lines represent the three directions (Anterior, posteromedial, and posterolateral) used in the analysis.

Postural control was quantified by reach distance in centimeter (cm) and the magnitude of the center of pressure (COP) movement and excursion in three directions (anterior, posteromedial and posterolateral) of the SEBT (30). A computerized force platform (SCIFIT Systems Inc., Tulsa, Oklahoma, USA) was used to acquire COP measures during the performance of the SEBT. The center of the SEBT grid was aligned with the center of the force plate (Fig 1).

#### Electromyography (EMG)

A 6-channel MyoMuscle 1200 EMG system (Noraxon USA, Inc, Scottsdale, AZ) with an input impedance of greater than 100 mΩ, a gain of 500, and a common-mode rejection ratio of greater than 100 dB was used to record muscle activity during the SEBT. The three chosen directions of the SEBT require sagittal and frontal plane stability. Thus, we recorded the activity of the hip and ankle muscles that contribute to sagittal plane stability (Gmax & TA) and those that contribute to frontal plane stability (Gmed & PL). EMG signals were acquired at a sampling rate of 1000 Hz.

### Procedures

#### Electrode placement

Subjects’ skin was shaved, abraded, and cleaned with isopropyl alcohol wipes prior to electrode placement. Surface electrodes (dual, 2 mm diameter, 2 cm apart, Noraxon USA, Inc) were placed parallel to the muscle fibers over the midsection of the muscle bellies in accordance with the SENIAM research group recommendations and previous research [31,32]. The Gmed electrode was placed one half of the distance between the iliac crest and the greater trochanter, while the Gmax electrode was placed midway between the second sacral vertebrae and the greater trochanter. TA electrode was placed at one-third the distance of a line between the head of the fibula and the medial malleolus, while PL electrode was placed on the line between the head of the fibula to the lateral malleolus, approximately 4cm distal to the fibular head. The same tester positioned all electrodes to maintain consistency. Electrodes and EMG sensors were further secured to the skin with an adhesive tape to prevent slippage during testing and minimize movement artifacts. Electrodes’ placement was confirmed by viewing EMG signals during a manual muscle test to minimize crosstalk between muscles.

#### MVIC evaluation

Prior to testing, subjects performed a 3-minute, submaximal warm-up on a stationary bicycle. For the Gmed testing, subjects were positioned in sidelying on the untested leg with the tested leg in a neutral position, supported by pillows between the lower extremities. The hip and knee of the untested leg were slightly flexed. For the Gmax testing, subjects were positioned in a half-pronelying position with both hips flexed to 90° while the knee of the tested leg in 90° of flexion and the opposite knee positioned in slight flexion. An immovable strap was placed around the lower thigh of the tested leg and the plinth to resist hip abduction and extension. A towel was placed between the strap and the subject’s leg for comfort. For testing, subjects produced a maximum voluntary isometric contraction (MVIC) using the make test [33]. They were instructed to avoid explosive contraction and to increase their effort gradually to their maximum once they hear the word “Go!”. Standard verbal encouragement was given during each trial. Subjects performed 1 practice sub-maximal contraction trial prior to the measurement trials to ensure adequate performance and stabilizations. Three 5-second measurement trials were completed for each muscle group with a 30-second rest period in between each trial. An additional trial was taken if more than 10% of variation was noted between trials to avoid large variability. The same tester performed all measurements to maintain consistency, and the order of muscle testing was randomized to account for any potential bias. Gmed and Gmax MVICs were collected to enable normalization of the EMG data.

#### SEBT protocol

Following MVIC testing, subjects had a 5-minute rest period. Afterwards, they were instructed to stand barefoot on the tested leg with their midfoot positioned over the center of a tape grid and slowly reach with the contralateral leg as far as possible, touch the line on the floor lightly with the tip of the foot of the reaching limb in three different directions [30] with respect to the stance limb (anterior, posteromedial, and posterolateral directions) while keeping the heel of the stance foot on the ground and their hands resting on their waist, then return to the starting position while maintaining single-leg stance balance for about 10 seconds before resting. Subjects were instructed to stand as motionless as possible during the last 10 seconds of single leg stance balance. Three practice trials in each reach direction were allowed to familiarize subjects with the test followed by three measurements trials. An additional practice trial was given when necessary. Thirty seconds of rest were given between each reach trial and 60 seconds between each direction to minimize fatigue. The test was demonstrated to each participant by one of the research team members prior to the practice trials. Subjects were verbally encouraged to reach as far as possible. A metronome was used at a rate of 60 beats/min to ensure consistent timing of each reach trial. The trial was discarded and repeated if subjects lifted the heel of the stance limb off the floor, did not keep their hands on their waist, touched down with their reach foot (weight bearing with the reaching limb), lost balance, or could not return to the starting position. The order of the reach directions was randomized to account for any potential bias. EMG and COP data were recorded simultaneously during the procedure.

### Data processing

#### Reach distance

During each trial, the examiner marked the point of maximal reach touched on the tape measure with an erasable ink and then manually measured the distance in cm from the center of the grid to each marked point with a tape measure. Measurements from the 3 trials were averaged and normalized to subject’s leg length, which was measured manually from the anterior superior iliac spine to the distal tip of the medial malleolus [34]. The average reach distance for each direction was expressed as a percentage of leg length and used for analysis.

#### COP data

COP measures were collected during the SEBT test including the COP excursion sway area (95% ellipse area), mean sway velocity, and path length. The area represented the magnitude of distribution of COP excursions during a trial, whereas velocity represented the average speed of COP movement during a trial. COP path length was the traveling distance of COP trajectory from the starting position to the maximal position of the COP during each trial. Data were collected during each reaching trial from the moment subjects lifted their limb until they returned to the starting position [35]. Data were recorded at 100Hz. Data collected from the three reaching trials in each direction was averaged and analyzed in respect of the average reaching distance within each direction.

#### EMG activation amplitudes

EMG signals were filtered at 10–500 Hz using a fourth order bandpass filter. All EMG data were then full wave rectified and smoothed using the root-mean-square algorithm with a 50-millisecond time constant. Peak amplitude was averaged over a 500 ms time window. For Gmed and Gmax, the highest peak value out of the three trials for each muscle was automatically selected, recorded as MVIC and used for normalization. The highest maximal voluntary contraction (MVC) of the TA and PL during all SEBT trials was used to normalize data between subjects [36]. To establish %MVIC and %MVC, peak amplitude value was calculated for each muscle during the period from toe off to touch down and return to starting position of each SEBT trial, which was determined visually. For each reaching direction, the peak value of each trial was normalized to the reference value (MVIC/MVC), expressed as percentage %MVIC/%MVC, and used for the analysis.

#### Muscle activation onset time

Muscle activation onsets were determined at the point of lifting the limb off the force plate. A muscle onset was defined as the point in which the EMG signal deviated by more than 2 standard deviations, for a minimum of 50 milliseconds (ms), above the baseline taken 100 ms prior to movement begin [37]. The onset of muscle activity determined by the algorithm was also visually confirmed to eliminate any potential movement artifacts that might be incorrectly identified as muscle activation onset. For each reaching direction, the average of the 3 trials of all muscles activation onset times was calculated and used for analysis.

### Statistical analyses

A sample size of 51 participants was estimated using a moderate effect size of 0.5, level of significance 0.05, and power of 0.80. We were able to recruit 48 participants, 16 in each group. Data was summarized using mean and standard deviation (SD) for quantitative variables and counts for qualitative variables. The normality of continuous variables was examined using Shapiro Wilk’s test. The distribution of subjects’ characteristics by study group was evaluated using chi-square for qualitative variables. We compared means of baseline quantitative variables among the study groups using One Way Analysis of Variance (ANOVA). Outcome variables were compared among groups using One Way ANOVA. Post hoc comparisons using Bonferroni test and Cohen’s d effect size were computed to identify specific differences when significant group main effects were detected. Effect size was calculated using GPower software (version 3.1.2, University of Dusseldorf, Dusseldorf, Germany). The level of significance was set at p≤0.05. All statistical tests were performed using IBM SPSS Statistics Software version 24 for Windows (Chicago, IL, USA).

## Results

A sample of forty-eight physically active individuals with mean age 27.7±4.5 years, height 171.0±7.9 cm, mass 73.2±12.9 kg, and body mass index 25.0±3.6 kg/m^2^ participated in this study. Subjects’ characteristics are summarized in Table 1. The distribution of all quantitative variables was approximately normal. There was no significant difference in characteristics of subjects by study group (p>0.05).

**Table 1:** Mean (SD) of baseline characteristics by study group (N = 48).

	CAI (n = 16)	Copers (n = 16)	Control (n = 16)	p-value
**Male (n)**	7	11	5	0.097
**Age, y**	29.6 (4.2)	27.8 (4.4)	25.8 (4.4)	0.059
**Height, cm**	170.2 (5.9)	172.1 (7.1)	170.8 (10.5)	0.805
**Mass, kg**	72.6 (16.9)	73.2 (9.6)	73.9 (12.1)	0.963
**BMI (kg/m**^**2**^**)**	24.9 (4.9)	24.6 (2.1)	25.3 (3.5)	0.874
**Leg length, cm**	89.6 (5.1)	90.7 (4.5)	88.6 (8.0)	0.601
**MD visit for LAS (n)**	11	7	n/a	0.143
**Grade of LAS (II/III, n)**	10/6	13/3	n/a	0.221
**LAS frequency (n)**	≥3 (16)	≤2 (16)	n/a	0.224
**Pain during sport (n)**	13	14	3	< 0.001
**Previous rehab (n)**	2	3	n/a	0.503
**Sport participation, hours per week**	5.8 (2.3)	6.4 (2.1)	7.3 (3.2)	0.207
**CAIT score**	16.3 (3.4)	28.1 (0.3)	29.4 (0.9)	< 0.001

**Abbreviation:** SD, Standard Deviation; CAI, Chronic Ankle Instability; BMI, Body mass index; MD, Medical Doctor; LAS, lateral ankle sprain; CAIT, Cumberland ankle instability tool; n/a, not applicable

### SEBT reach distance

Results are displayed in Table 2. There was a significant difference in mean reach distance during the anterior direction (AD) among the three study groups (p = 0.019, η^2^ = 0.37). When compared to copers and controls, CAI group had significantly less reach distance during the AD (p = 0.021, η^2^ = 0.30 and p = 0.009, η^2^ = 0.35, respectively). However, no significant difference was found between copers and controls in the AD (p = 0.354, η^2^ = 0.04). For the other two reach directions, posteromedial direction (PMD) and posterolateral direction (PLD), there was no significant difference in mean reach distance among the study groups (p>0.05).

**Table 2:** Mean (SD) of postural control by reach direction among study group.

	Reach Direction	CAI (n = 16)	Copers (n = 16)	Control (n = 16)	*F* value	*p* value*	Effect size (η^2^)
**Reach Distance, %**	Anterior	82.1 (7.8)	89.1 (7.6)	90.1 (12.4)	4.0	0.019	0.38
	Posteromedial	90.8 (8.0)	93.9 (8.5)	95.4 (10.8)	1.2	0.177	0.21
	Posterolateral	82.0 (15.6)	85.4 (12.0)	86.9 (12.0)	0.5	0.304	0.15
**Sway Velocity (mm/sec)**	Anterior	70.5 (16.3)	60.6 (10.7)	55.4 (19.5)	3.7	0.016	0.40
	Posteromedial	73.8 (23.4)	66.0 (18.7)	58.6 (18.7)	2.2	0.049	0.31
	Posterolateral	75.5 (15.9)	65.8 (14.6)	56.4 (16.8)	5.9	<0.01	0.50
**95% Confidence Ellipse Area (mm^2^)**	Anterior	3602.9 (1789.0)	3419.7 (1176.4)	2897.6 (1215.7)	1.2	0.177	0.21
	Posteromedial	2998.7 (1803.4)	2767.1 (1238.4)	2372.5 (979.1)	0.8	0.219	0.19
	Posterolateral	2321.0 (902.1)	2330.4 (1373.8)	1998.9 (660.5)	0.5	0.292	0.15
**Path Length (mm)**	Anterior	977.9 (245.9)	844.1 (148.2)	767.3(282.6)	3.4	0.022	0.37
	Posteromedial	1011.6 (329.0)	920.6(234.0)	823.7 (260.2)	1.8	0.085	0.28
	Posterolateral	1037.2 (247.7)	904.0(169.1)	791.3 (203.2)	5.5	<0.01	0.50

**Abbreviation:** SD, Standard Deviation; CAI, Chronic Ankle Instability

For all variables except reaching distance, lower scores indicate better postural stability

*One-way analysis of variance (ANOVA); level of significance was set at *p* ≤0.05

### COP sway velocity

Results are summarized in Table 2. Examining each direction separately, a significant difference was found among the study groups during AD (p = 0.016, η^2^ = 0.40), PMD (p = 0.049, η^2^ = 0.31), and PLD (p<0.01, η^2^ = 0.50). Post hoc comparison for the AD showed that CAI group had a significant higher mean COP sway velocity as compared to controls (p<0.01, η^2^ = 0.39) and copers (p = 0.041, η^2^ = 0.25); however, no significant difference was detected between copers and controls (p = 0.179, η^2^ = 0.13). For PMD, CAI group also had a significant higher mean COP sway velocity when compared to controls (p = 0.020, η^2^ = 0.32), but no significant difference was found between CAI and copers (p = 0.141, η^2^ = 0.16) or between copers and control (p = 0.155, η^2^ = 0.16). For the PLD, CAI group displayed a significantly higher mean COP sway velocity as compared to controls (p<0.001, η^2^ = 0.50) and copers (p = 0.045, η^2^ = 0.25). Likewise, copers had a significant higher mean COP sway velocity as compared to controls during the PLD (p = 0.049, η^2^ = 0.24).

### COP sway area

Results are presented in Table 2. There was no significant difference in mean COP 95% confidence ellipse area during all directions among the three study groups (p>0.05, η^2^ = 0.18).

### COP path length

Results are displayed in Table 2. There was a significant difference in mean COP path length during AD (p = 0.022, η^2^ = 0.37) and PLD (p<0.01, η^2^ = 0.50) among the study groups. Post hoc comparison of the AD showed that CAI group had a significantly higher mean COP path length as compared to controls (p<0.01, η^2^ = 0.37) and copers (p = 0.049, η^2^ = 0.23); however, no difference was found between copers and controls (p = 0.177, η^2^ = 0.13). Similarly, in the PLD, CAI group displayed significantly increased mean COP path length when compared to controls (p = 0.001, η^2^ = 0.50) and copers (p = 0.039, η^2^ = 0.26); however, no difference was found between copers and controls (p = 0.068, η^2^ = 0.22).

### EMG activation amplitudes

Results for the SEBT EMG activity in the anterior, posteromedial, and posterolateral directions are summarized in Table 3. There was a significant difference in mean EMG activity of TA during AD (p<0.01, η^2^ = 0.71) and PL and Gmax during PLD (p = 0.049, η^2^ = 0.32 and p = 0.011, η^2^ = 0.50, respectively) among the three study groups. When compared to copers and controls, CAI group had significantly less TA activity during AD (p = 0.013, η^2^ = 0.44 and p<0.01, η^2^ = 0.70, respectively). During PLD, the CAI group had less Gmax activation than controls (p<0.01, η^2^ = 0.43) and copers (p = 0.019, η^2^ = 0.35). Also, copers had higher activation in PL as compared to controls during PLD (p = 0.039, η^2^ = 0.31). However, there were no other significant differences identified between the groups for the other directions.

**Table 3:** Mean (SD) of EMG activation by reach direction among study group.

Muscles	Reach Direction	CAI (n = 16)	95% CI	Copers (n = 16)	95% CI	Controls (n = 16)	95% CI	*F* value	*p* value*	Effect size (η^2^)
**Tibialis Anterior (%MVC)**	Anterior^a^	33.1 (10.1)	38.6, 47.7	44.8 (13.4)	37.7, 51.9	51.7 (8.4)	47.2, 56.1	12.1	<0.01	0.71
	Posteromedial	56.2 (11.0)	50.4, 62.0	52.9 (9.1)	48.1, 57.8	59.1 (8.8)	54.4, 63.8	1.6	0.104	0.26
	Posterolateral	57.3 (9.7)	52.1, 62.5	60.9 (4.8)	58.3, 63.4	60.7 (11.0)	54.8, 66.6	0.8	0.230	0.18
**Peroneus Longus (%MVC)**	Anterior	44.8 (11.8)	38.6, 51.1	51.7 (7.5)	47.7, 55.7	46.2 (13.8)	38.9, 53.6	1.6	0.103	0.26
	Posteromedial	49.0 (10.0)	43.7, 54.3	52.3 (9.9)	47.5, 58.1	49.0 (9.6)	43.9, 54.2	0.8	0.231	0.16
	Posterolateral^b^	51.0 (10.7)	45.3, 56.7	57.2 (10.2)	51.8, 62.6	48.7 (13.0)	41.7, 51.6	2.4	0.049	0.32
**Gluteus Maximus (%MVIC)**	Anterior	15.3 (7.7)	11.2, 19.4	19.7 (11.4)	13.6, 25.8	21.1 (14.4)	13.4, 28.8	1.1	0.174	0.21
	Posteromedial	38.0 (13.0)	31.0, 44.9	41.2 (16.1)	32.6, 49.8	44.0 (17.4)	34.8, 53.3	0.6	0.277	0.16
	Posterolateral^c^	25.6 (9.4)	20.6, 30.6	37.5 (13.8)	30.1, 44.8	40.2 (17.2)	31.0, 49.3	5.3	0.011	0.50
**Gluteus Medius (%MVIC)**	Anterior	28.3 (12.9)	21.4, 35.2	28.2 (10.9)	22.4, 34.0	27.0 (13.7)	19.7, 34.3	0.1	0.475	0.05
	Posteromedial	53.2 (11.8)	47.0, 49.4	46.2 (11.8)	39.9, 52.5	49.2 (14.4)	41.6, 56.9	1.2	0.151	0.23
	Posterolateral	39.7 (11.1)	33.8, 45.6	45.9 (11.3)	39.9, 51.8	42.0 (13.4)	34.8, 49.1	1.1	0.172	0.21

**Abbreviation:** SD, Standard Deviation; CAI, Chronic Ankle Instability; CI, Confidence Interval; MVC, Maximal

Voluntary Contraction; MVIC, Maximal Voluntary Isometric Contraction

^a^ Significant difference between CAI and controls (p<0.01) and between CAI and copers (p = 0.013)

^b^ Significant difference between copers and controls (p = 0.039)

^c^ Significant difference between CAI and controls (p<0.01) and between CAI and copers (p = 0.019)

*One-way analysis of variance (ANOVA); level of significance was set at *p* ≤0.05

### Muscle activation onset time

**Fig 2:**
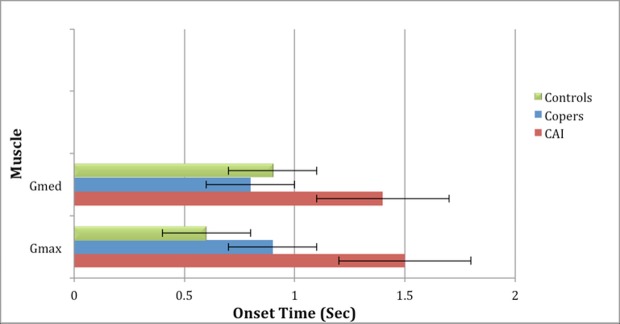
Mean onset time in seconds of gluteus maximus—Gmax (anterior direction) and gluteus medius—Gmed (posteromedial direction) among study groups.

There was a significant difference in mean Gmax muscle activation onset time (seconds) among CAIs, copers and controls during the AD (1.5 ± 0.4 vs. 0.9 ± 0.2 vs. 0.6 ± 0.2, p = 0.025, Fig 2). The difference was statistically significant between CAI and control groups (p = 0.031). There was also a significant difference in mean Gmed muscle activation onset time (seconds) among CAIs, copers and controls during the PMD (1.4 ± 0.3 vs. 0.8 ± 0.2 vs. 0.9 ± 0.2, p = 0.038, Fig 2). The difference was statistically significant between CAI and copers groups (p = 0.035).

## Discussion

To the best of the authors’ knowledge, this is the first study that simultaneously examined postural control and EMG activation of the ankle and hip muscles during the performance of the SEBT in individuals with and without CAI. We identified group differences in postural control measures and EMG activity during the performance of SEBT. The CAI group demonstrated diminished dynamic postural control, delayed muscle activation onset times, and less activity of the muscles acting on the ankle and hip than the other groups. No significant differences were observed between copers and controls except for mean COP sway velocity and PL activation in the PLD.

### Postural control

The analysis of outcome measures revealed that patients with CAI exhibited poor postural control performance as demonstrated by reach distance and COP measures when compared to healthy individuals. Patients with CAI had significantly less reach distance in the AD as compared to the other groups. Similar findings were reported by previous researchers [21,22,30]. In our study, however, we found no significant differences in the PMD reach distance among groups, and this was in line with the results reported by Pozzi et al [38]. The PMD was reported to be “most representative” of all other directions in the SEBT [30]; however, it appears that using this direction alone might not be sensitive enough to show differences between stable and unstable ankles. Thus, a combination of more than one direction such as the Y balance test, which consists of three directions (AD, PMD and PLD), may be more applicable.

Postural control was also quantified by the magnitude of the COP movement and excursion during the performance of the SEBT. Though the SEBT has been shown to be sensitive in detecting dynamic postural control deficits associated with CAI [21,23,24], we anticipated that relying on the reach distance alone might not be sensitive enough to detect differences, especially with the controversial findings reported in previous research [38]. Reach distance was only significantly different in the AD, however, COP measures showed many significant differences among the groups in most of the directions, suggesting that relying on the reach distance alone might not provide the full picture of the postural control deficits if they are indeed present. The ability to maintain good stability while reaching is essential. An individual with an unstable ankle might be able to complete a functional task as well as a person with stable ankle depending on the severity of his/her condition; however, the completion pattern might be altered, creating the potential threat of reinjury. In this study, CAI patients were able to reach as far as healthy subjects, however, they exhibited a higher sway as shown by the COP measures, which implies an impaired postural control. Though significant differences in COP measures were identified during the AD (η^2^ = 0.4), the PMD (η^2^ = 0.3), and PLD (η^2^ = 0.5), in this study, PLD showed to be more challenging for CAI patients. During testing, it was observed that most of the participants had difficulty maintaining stability when reaching in the PLD even after practice trials were given.

### Neuromuscular control

Altered NMC patterns in proximal and distal joint muscles have been previously identified in patients with CAI during functional tasks [8,9,16,17,18]. Patients with CAI have demonstrated a delay in hamstring, Gmax (bilaterally), and erector spinae activation during a prone hip extension testing when compared to healthy controls [39]. CAI patients also have exhibited decreased ankle and hip muscle activity during the performance of functional rehabilitative exercises [16,17]. Similar to our findings, when compared to controls, patients with CAI had reduced TA activity during the AD of the SEBT, with no differences reported for the Gmed activity [17]. However, muscle activation onset times and Gmax activity were not recorded in that study. CAI patients in the present study had significantly delayed gluteal muscles activation onset times during the AD and PMD and had less Gmax activity during PLD of the SEBT. This further supports the findings reported by Webster and Gribble [16] during a single leg rotational squat exercise, which were considered as a potential factor for the continual instability. Reaching in the PLD is especially challenging, as individuals have to maintain a level pelvis on the stance leg. As individuals reach backward across the stance leg, they shift their trunk anteriorly to maintain the center of mass within the base of support. Flexion in the trunk produces flexion moment at the hip, which is controlled by contraction of the hip extensors [40]. Thus, the elevated activation of Gmax might be needed in this situation to counteract the sagittal plane flexion of the trunk and hip. It seems that patients with CAI did not fire the Gmax enough to counteract this motion, resulting in overcompensation to maintain the body’s center of mass within the base of support, which may have led to the higher sway during the PLD. The higher activation in the Gmax might also have occurred in order to control for the internal rotation of the femur during the PLD. These explanations, however, are hypothetical given the fact that kinematic data were not examined in the present study. The turning or twisting movement such as that in the PDL is crucial to athletic activity and is usually a common mechanism of lower extremity injuries [16].

Nonetheless, in the above studies [16,17], instrumented postural control data was not collected. We collected postural control and EMG data simultaneously during the performance of the SEBT to examine potential sources of performance deficits and to further understand the NMC strategies utilized by each group to maintain stability. In this study, CAI group demonstrated poorer performance mainly during the AD and PLD of the SEBT. Interestingly, during both directions, CAI group had less activation in the TA and Gmax than the other groups, which might indicate a relationship between performance and altered NMC at the hip and ankle. The higher activation seen in controls and copers could be interpreted as a strategy used by these individuals to maintain stability where the task is more challenging. Thus, the poorer performance noted in the CAI group during these directions could be attributed to the lack of such strategy. Furthermore, it was noted that during the PLD, controls and copers used both ankle and hip muscles, which was not the case for the CAI group who relied more on the ankle muscles to complete the task. In addition, significantly delayed gluteal muscles activation onset times were observed for the CAI group, while the onset times of the ankle muscles were not significantly altered. This reliance on ankle strategy to maintain stability might be considered a suboptimal muscle recruitment pattern and it could explain the increased risk of injury in this population. Gribble and Hertel [41] previously reported that fatigue to the proximal musculature of lower extremity created significantly increased postural control deficits compared to fatigue of the distal muscles. However, we identified alterations in the proximal and distal joint muscles activity in CAI patients without administrating a fatiguing protocol, which might be responsible for the altered postural control in this population.

During weight bearing activities, the muscles around the hip work to maintain pelvis stability and control the movement of the femur, which subsequently affects positioning of the ankle and foot [16,42]. This control of the pelvic motion is critical to maintain total body stability [43]. Small errors in balance are usually corrected distally by the musculature of the foot and ankle, whereas large errors are rectified at the hip [43]. Absence of a good motor control at the hip joint might increase the workload on the ankle musculature in order to maintain stability, which may lead to future episodes of injury. Moreover, patients with CAI showed decreased activity of the ankle muscles, and this could further affect the stability of the ankle complex.

While previous studies [10,11,38] reported copers had higher activation in TA and PL than CAIs and controls, in the present study, copers displayed greater activation of the TA (AD) and Gmax (PLD) and earlier gluteal muscles activation onset timing (AD, PMD) as compared to CAIs but not to the controls and had higher activation of PL (PLD) as compared to controls only. The different testing protocols implemented by the studies might have led to such dissimilarity. However, it is unknown whether these NMC strategies exhibited by the copers were already present before injury or developed after the injury as a successful compensatory mechanism against instability. Irrespective of that, such strategies might help copers to maintain postural stability as indicated by our results and therefore minimize the risk of developing instability. The delayed and diminished activation may suggest that CAI patients may not have fully developed this coping mechanism, which puts them at higher risk of reinjury and instability. Furthermore, perhaps the time allowed for healing after incurring an initial injury prior to return to sports might has an effect on the level of neuromuscular adaptations. Future research should examine whether time and process of healing after an initial sprain is associated with ankle instability. Examining postural control early after acute lateral ankle sprain could also provide information about the neuromuscular adaptations acquired by this population.

It is worth mentioning, however, that the mean age of the population included in this study was between 25 and 30 years of age, which was higher than the age reported (20–21 years) in the majority of the previous CAI research. It might be possible that we were not able to identify more differences in the outcome variables between groups because these patients have had longer time to mature and adapt motor strategies based on their impairments due to their age and how long they have been experiencing CAI symptoms.

Traditionally, there has been a focus on the distal ankle musculature, mainly the peroneal, for their capacity to provide dynamic stability and for the goal of preventing subsequent ankle injuries. Interestingly, the study results showed an alteration in both, the distal and proximal musculature activity in patients with CAI. These findings substantiate that deficits associated with CAI may be the consequences of both peripheral and centrally mediated alterations in NMC [13–17]. However, examining the contralateral limb to determine if there were subtle alterations in sensorimotor control would further add to these findings [13]. Hip musculature plays a major role in maintaining postural stability during single leg activities such that in SEBT. Clinically, this is important, as single leg activities are a key component of almost all functional movements. Alteration in the proximal muscles activity appears to negatively affect measures of postural control and the quality of movement, which may lead to prolonged functional impairments and increased recurrence of the undesired lower extremity injuries in this population. Overall, with the diminished hip and ankle muscles activation, the body’s ability to maintain postural stability is compromised, thus the ability to perform functional skills might be limited in this population.

### Study limitations

Limitations of this study include the lack of kinematic data to further support the findings of this study, the number of muscles recorded, and the lack of measuring dorsiflexion range of motion (ROM). Subjects with CAI were reported to have a dorsiflexion ROM deficit [44], which might have an effect on the overall performance, mainly reaching in the anterior direction. In addition, since most injuries occur unpredictably, including an unexpected perturbation to the SEBT may provoke different NMC strategies and therefore should be examined. Finally, the smaller sample size with large standard deviations may have resulted in a type II error when statistical significance was not noted. In addition, post hoc power analysis using the effect size of 0.4 that was found in the reach distance, sway velocity, and path length in the AD, revealed that the power was 0.70. Thus, future research with a larger sample size that examines muscles activity and balance during more functional tasks such as running is needed.

### Implications for practice

Findings from this study have provided additional insight regarding the NMC deficits in this population. The authors’ current data support that clinicians, in addition to examining ankle joint function, should also examine and address hip impairments for the treatment of CAI. Interventions should emphasis on relearning coordinated and multi-segmental anticipatory neuromuscular strategies to provide an effective outcome. Incorporating exercises with a special focus on the inclusion of hip muscles recruitment into the rehabilitation program can improve hip muscles activity and dynamic postural control, which may help to reduce the risk of reinjury and improve functional performance in this population. Another important point to consider is that the PLD was found to be more challenging for CAI patients. This could be important for clinicians to use when examining postural control in patients with CAI or when using the SEBT as an intervention to improve postural control. However, using a combination of more than one direction such as the Y balance test is recommended in a clinical setting to detect such problems.

## Conclusion

We were able to identify alterations in proximal and distal NMC in patients with CAI. These alterations appear to negatively affect measures of postural control in this population. Overall, patients with CAI exhibited poorer postural control and diminished hip and ankle muscles activity. However, caution should be taken when interpreting these findings, since it is not known whether these alterations are the cause of or a result of CAI. In addition to ankle muscles activity, improving hip muscles activity might help the body to produce functional movements while maintaining pelvis stability. Thus, targeting hip muscles in the conditioning and rehabilitation program might benefit this population.
